# Draft Genome Sequence of an Epiphytic Strain, *Bacillus* sp. Strain WL1, Isolated from the Surface of Nostoc flagelliforme Colonies in Yinchuan, Ningxia, China

**DOI:** 10.1128/MRA.00382-21

**Published:** 2021-07-29

**Authors:** Xiang Gao, Tao Zheng, Xiaolong Yuan, Zhengke Li, Yongjun Wei

**Affiliations:** a School of Food and Biological Engineering, Shaanxi University of Science and Technology, Xi’an, Shaanxi Province, China; b School of Life Sciences, Central China Normal University, Wuhan, Hubei Province, China; c Laboratory of Synthetic Biology, Key Laboratory of Advanced Drug Preparation Technologies, Ministry of Education, School of Pharmaceutical Sciences, Zhengzhou University, Zhengzhou, Henan Province, China; University of Maryland School of Medicine

## Abstract

*Bacillus* species are Gram-positive, aerobic, spore-forming bacteria that are widely spread in soil, dust, and water. One strain, *Bacillus* sp. strain WL1, was isolated from the surface of the cyanobacterium Nostoc flagelliforme in Yinchuan, Ningxia, China. The draft genome sequence of this strain was 5.62 Mbp.

## ANNOUNCEMENT

Nostoc flagelliforme is a terrestrial blue-green alga that is distributed throughout the arid and semiarid steppes of the western and northwestern parts of China (1). It exhibits a predominantly filamentous colony shape. It was previously reported that the epiphytic bacteria of *N. flagelliforme* were mainly *Bacillus* spp., including Bacillus subtilis, B. licheniformis, B. megaterium, B. cereus, and B. circulans. In this study, we attempted to isolate and identify those epiphytic bacteria on the surface of *N. flagelliforme* collected on the eastern side of Helan Mountain in Yinchuan, Ningxia, China (2). The epiphytic bacteria were washed from the *N. flagelliforme* filaments with sterilized water and cultivated with Luria-Bertani (LB) solid medium at 37°C. One dominant strain with a uniform shape and size was isolated. The 16S rRNA gene was amplified with the primers 27F (5′-AGAGTTTGATCCTGGCTCAG-3′) and 1492R (5′-GGTTACCTTGTTACGACTT-3′) as previously described (3). This strain was identified as a *Bacillus* sp. based on 16S rRNA gene sequencing and named *Bacillus* sp. strain WL1.

*Bacillus* sp. strain WL1 was stored in glycerol solution (20%, vol/vol) at −80°C. The strain was streaked onto solid LB medium and cultivated at 37°C. One single *Bacillus* sp. strain WL1 colony was inoculated into liquid LB medium for shake cultivation overnight at 30°C, and the cell culture was collected by centrifugation for DNA extraction using a DNeasy PowerMax soil kit (Qiagen, Germany) (4). The DNA was used to build a 2 × 150-bp sequencing library and sequenced using the Illumina HiSeq 2500 platform at Genesky Bio-Tech Co., Ltd. (Shanghai, China); the raw reads of *Bacillus* sp. strain WL1 were filtered using Trim Galore v0.4.4 (http://www.bioinformatics.babraham.ac.uk/projects/trim_galore/). The total number of reads from the paired-end sequencing (2 × 150 bp) was 4,372,767, and a total of 658.84 Mbp of data was obtained. The sequence coverage of this genome was 117×. The draft genome sequences were assembled using SOAPdenovo v2.04 with default parameters, and the resulting assembly was used for further genomic analysis (5, 6). The gaps in the draft genome sequence were corrected using GapCloser v1.12 software (6). RNAmmer v1.2 and tRNAscan-SE v2.0 were used for rRNA and tRNA prediction, respectively (7, 8). Glimmer3 v3.02 (http://ccb.jhu.edu/software/glimmer/index.shtml) was used for gene prediction. Default parameters were used for all software unless otherwise specified.

A total of 420 contigs comprised the draft genome assembly of *Bacillus* sp. strain WL1. The total length of the draft genome sequence is 5,624,893 bp, with a GC content of 35.2%. The length of the largest contig is 226,802 bp, and the *N*_50_ contig length is 38,057 bp. A total of 5,697 genes are predicted in the draft genome sequence of *Bacillus* sp. strain WL1, and the average gene length is 804.9 bp. The gene length composed 81.1% of the whole draft genome sequence. The public version of the draft genome was annotated using PGAP v5.1 through NCBI (9), and the annotated genome sequence was deposited in the GenBank database (9). Genome-based phylogenetic analysis suggested that this strain was most similar to B. cereus ATCC 14579.

### Data availability.

The draft genome sequence of *Bacillus* sp. strain WL1 was deposited in GenBank under accession number JAGKST000000000, BioProject accession number PRJNA733760, BioSample accession number SAMN19458293, and SRA accession number SRR14099160.
